# Influence of Size Effect in Milling of a Single-Crystal Nickel-Based Superalloy

**DOI:** 10.3390/mi14020313

**Published:** 2023-01-26

**Authors:** Luis Soriano Gonzalez, Fernanda Medina Aguirre, Sein Leung Soo, Richard Hood, Donka Novovic

**Affiliations:** 1 Machining Research Group, Department of Mechanical Engineering, School of Engineering, University of Birmingham, Edgbaston, Birmingham B15 2TT, UK; 2 Manufacturing Technology, Rolls-Royce Plc, More Lane, Derby DE24 8BJ, UK

**Keywords:** micromilling, single-crystal nickel-based superalloys, minimum uncut chip thickness, size effect

## Abstract

This paper details an experimental investigation on the influence of the size effect when slot-milling a CMSX-4 single-crystal nickel-based superalloy using 1 mm- and 4 mm-diameter TiAlN-coated tungsten carbide (WC) end-mills. With all tools having similar cutting-edge radii (r_e_) of ~6 µm, the feed rate was varied between 25–250 mm/min while the cutting speed and axial depth of cut were kept constant at 126 m/min and 100 µm, respectively. Tests involving the Ø 4 mm end-mills exhibited a considerable elevation in specific cutting forces exceeding 500 GPa, as well as irregular chip morphology and a significant increase in burr size, when operating at the lowest feed rate of 25 mm/min. Correspondingly for the Ø 1 mm micro-end-mills, high levels of specific cutting forces up to ~1000 GPa together with severe material ploughing and grooving at the base of the machined slots were observed. This suggests the prevalence of the size effect in the chip formation mechanism as feed per tooth/uncut chip thickness decreases. The minimum uncut chip thickness (h_min_) when micromilling was subsequently estimated to be less than 0.10 r_e_, while this increased to between 0.10–0.42 r_e_ when machining with the larger Ø 4 mm tools.

## 1. Introduction

Owing to the absence of grain boundaries coupled with tailored chemical compositions to increase high-temperature strength, single-crystal (SX) nickel-based superalloys possess remarkable fatigue- and creep-resistance properties, which make them suitable for critical rotating and stationary parts in the hot sections of aeroengines such as turbine blades and nozzle guide vanes. Such components often incorporate complex geometries as well as small features, which are subjected to tight dimensional tolerances [1], together with stringent surface integrity standards to meet functional and reliability requirements. The manufacturing of these components is increasingly challenging, especially with the growing trend towards miniaturisation to reduce engine weight and enhance bypass ratios, which are necessary for lowering operational costs and fuel consumption/emissions [2]. Microscale milling has been identified as a potential production technology for such applications, due to its capability to achieve superior material-removal rates, dimensional accuracy and surface quality compared to alternative solutions based on electrochemical, electro-physical and photonic processes [3]. Furthermore, with recent developments in additive manufacturing (AM) technologies for producing advanced nickel-based superalloys including single crystals [4,5], it is anticipated that micromilling will be a key process in the future for addressing the geometrical accuracy and surface roughness limitations inherent in workpieces/parts fabricated using AM processes [6].

While there has been rising interest in micromilling research over the past decade, the majority of studies involving metallics have focused on materials such as steels, copper and aluminium alloys [7]. In contrast, the published literature relating to the machining of single-crystal nickel-based superalloys using small-diameter cutting tools remains limited [8]. According to Câmara et al. [9], micromilling can be defined based on the diameter of the cutting tool when in the region of 1–1000 µm, but they emphasise that a more important distinguishing factor is that the undeformed/uncut chip thickness (h) tends to be in the order of the tool cutting-edge radius (r_e_) and grain size of the workpiece material. Under such conditions, the tool engages at a negative rake angle, leading to compression and ploughing of the workpiece without effective shearing/removal of material unless the specified depth of cut or feed per tooth (f_z_) is at least equivalent to a critical undeformed chip thickness value, which is defined as the minimum chip thickness (h_min_). It has been reported that h_min_ generally lies between 0.20–0.40 of the r_e_ for a range of polycrystalline metallic alloys [10]. This characteristic behaviour of unstable chip formation where elastic deformation/ploughing is prevalent instead of the traditional shearing mechanism (in conventional macro-scale machining), is caused by and described as the size effect [11]. It is also usually accompanied by a rapid/sharp increase in specific cutting forces, particularly as the uncut chip thickness is reduced, which can lead to detrimental workpiece surface integrity [12]. Furthermore, arbitrarily increasing the uncut chip thickness to improve cutting efficiency during micromilling is often not feasible, as the risk of higher cutting loads can easily lead to catastrophic edge fracture or tool breakage [13]. Therefore, understanding the incidence of the size effect is essential for selecting appropriate cutting conditions as well as for optimising micromachining operations. Other factors that can potentially influence machining performance are physiochemical and physical-based surface effects, which were recently reviewed by Lee and Wang [14]. While several studies have demonstrated that the physiochemical Rehbinder effect (reduction in material strength/ductility/hardness due to adsorption of a surfactant) can arise during machining, resulting in lower cutting forces and changes in the chip deformation mechanism, many of the reported experiments utilised surface-active media that are not generally representative of the compositions in metalworking fluids [14]. Additionally, the replicability of the Rehbinder effect and its degree of influence in comparison to machining size effects, particularly in the microcutting length scale, have not been definitively verified. Similarly, physical-based surface effects such as workpiece surface coating and extrusion cutting are not relevant in the present study.

Due to the complexity of micromilling, the exact influence of the size effect on process mechanics and the resulting workpiece surface integrity is difficult to ascertain, as this depends on several factors including tool cutting-edge geometries, workpiece microstructure and material properties, as well as cutting conditions/parameters [12,15]. However, the variation in specific cutting energy/force (k_c_) with respect to the ratio of feed per tooth to cutting-edge radius (f_z_/r_e_) can normally be utilised as an indicator of size effect initiation [16]. When micromilling maraging steel, Yao et al. [17] detected an increase in specific cutting energy of up to 300% as the f_z_/r_e_ ratio decreased from 1 to 0.125. In addition, a steep escalation in workpiece surface roughness was recorded as the chip load decreased below an f_z_/r_e_ ratio of 0.5. Aramcharoen and Mativenga [18] also reported the exponential growth of specific cutting forces and surface roughness when the f_z_/r_e_ ratio was lower than 1.0 during micromilling trials on H13 tool steel. Similarly, Vipidas et al. [19] observed a non-linear rise in the measured resultant cutting forces and tool–chip coefficient of friction at f_z_/r_e_ < 0.3, when machining Ti-6Al-4V using 1.0 mm-diameter WC end-mills with an r_e_ of ~3–4 µm, whilst an increase in surface roughness from 0.08 to 0.21 µm Ra was obtained as the f_z_/r_e_ ratio decreased ten-fold from 0.67 to 0.067, which was attributed to the elastic recovery of the material following ploughing.

The influence of the size effect can also be identified from the variation in cutting force response, as highlighted by Chen et al. [20]. An analysis of the thrust and cross-feed cutting force signals resulting from microgrooving tests showed highly irregular patterns, comprising only two maximum peaks with microfluctuations during two complete tool revolutions when f_z_/r_e_ was 0.5. This led to side flow/material drag marks on the machined surface. As an alternative to the commonly used force signatures, Mian et al. [21] utilised acoustic emissions (AE) together with wavelet and fast Fourier transform (FFT) processing techniques to identify energy bands related to deformation mechanisms in a variety of metallic workpiece materials subjected to micromilling. Based on the AE signatures, the minimum uncut chip thickness relative to cutting-edge radius was found to be within 11–18%, 33–39%, 31–42%, 20–36%, 19–28% and 19–26% for oxygen-free high-conductivity (OFHC) Cu, Al 6082-T6, AISI 1005 and 1045 steels, Ti-6Al-4V and Inconel 718, respectively.

Due to the intrinsic characteristics of the process, chips generated in micromilling are typically no longer than a few hundred micrometres, making them difficult to collect and evaluate [22]. Nonetheless, the investigation of chip formation is important as it can assist in correlating the influence of the size effect with the mechanics of microscale cutting [23]. De Oliveira et al. [10] conducted experimental trials on AISI 1045 steel employing micro-end-mills (Ø 0.8 mm) with a cutting-edge radius of 2.74 µm at varying f_z_ (0.1–7.0 µm/tooth). Here, the h_min_ was found to be within 0.6–1.0 µm (0.22–0.36 r_e_), as this resulted in a transition from needle-shaped chips to curled chips with a well-defined lamellae structure. Below the h_min_, an exponential increase in specific cutting forces was recorded together with ploughing marks on the machined surface. The influence of the size effect on the chip formation when machining P-20 die steel was investigated by Sahoo et al. [24]. Similarly, the h_min_ was determined by considering the changes in chip morphology when varying the f_z_/r_e_ ratio. Helical and curled chips were formed when machining above the h_min_ (f_z_/r_e_ > 0.33), whilst irregular-shaped swarf were obtained below this threshold. During the micro-slot-milling of Zr-based bulk metallic glass, Liu et al. [25] reported longer and more consistently formed chips when increasing the feed (0.02–0.06 µm/tooth) and decreasing the cutting speed (566–189 m/min). Using custom-developed Ti(C_7_N_3_)-based cermet end-mills (~4.13 µm r_e_ and ~990 µm diameter), Xu et al. [26] evaluated the chip morphology obtained at different feeds (0.5, 1.0, 1.5, 2.0 µm/tooth) during micro-slot-milling tests on 2024 aluminium alloy. At the lowest f_z_, extensive fractures were seen along the chip root, with the resulting width found to be ~20% shorter than the depth of cut (79 vs. 100 µm), suggesting an unstable chip formation mechanism dominated by ploughing.

To date, investigations addressing the mechanics and minimum chip thickness in the microcutting of Ni-based superalloys have predominantly involved polycrystalline materials. De Oliveira et al. [27] analysed the chip morphology after the micromilling of Inconel 718 using 400 µm-diameter end-mills, and identified the formation of helical chips with lengths comparable to the width of the slots when the main cutting edge was effectively shearing the material. In contrast, the significant compressive and shear stresses (2600 MPa) stemming from the engagement of the minor cutting edge with the workpiece, promoted the development of longer ribbon-shaped chips generated over consecutive revolutions together with spheroid swarf due to a higher friction at the tool–workpiece interface. In a study involving the micromilling of Inconel 718, Mian et al. [28] correlated the periodic variation in AE signals to the frequency of chip serration, and identified a shift from plastic deformation to microfracture with higher amplitudes of AE as the cutting speed increased from 10 to 40 m/min when f_z_/r_e_ was fixed at 0.4. To study the material-removal mechanism related to micromilling, Alhadeff et al. [22] collected chips produced from slot-milling trials on Hastelloy C-276 using 500 µm-diameter, AlTiN-coated WC end-mills at an f_z_ of 0.8 µm/tooth. Serrated chips were obtained, consisting of localised intense shear bands followed by relatively undeformed regions, which were attributed to high cutting temperatures. In addition, it was highlighted that the workpiece grain size (~10 µm) relative to the tool diameter is a major consideration in micromilling, as the material is effectively inhomogeneous under such conditions. This led to significant fluctuations in cutting forces resulting in the abrupt fracture of the cutting edges.

The microstructure of the workpiece can have a considerable impact on the initiation of the size effect in micromilling. Polycrystalline metallic alloys with grain sizes comparable to the undeformed chip thickness generally require higher specific energies for material removal, as shear and slippage do not necessarily progress along the weaker grain boundaries. Instead, material deformation upon engagement with the cutting tool predominantly occurs within the grain, inducing higher mechanical stresses [7,29]. For single-crystal alloys, the mechanism of grain boundary sliding does not arise [29]. Here, the motion of dislocations within the material is directly dependent on the lattice structure (unit cell) of the alloy [30], which can influence the onset of the size effect. However, research pertaining to the micromachining of single-crystal Ni-based superalloys has hitherto mainly focused on aspects such as the optimisation of cutting parameters and conditions for improving hole quality in microdrilling [29,31], as well as the assessment of cutting forces [32], tool wear mechanisms [33] and surface quality [34] following micromilling operations. Therefore, the present work aims to investigate the influence of the size effect on chip formation, tool wear, cutting forces and surface quality when slot-milling a single-crystal Ni-based superalloy using micro- (Ø 1 mm) and conventional/macro-sized (Ø 4 mm) end-mills, with the results subsequently analysed to estimate the h_min_ value.

## 2. Materials and Methods

**Figure 1:**
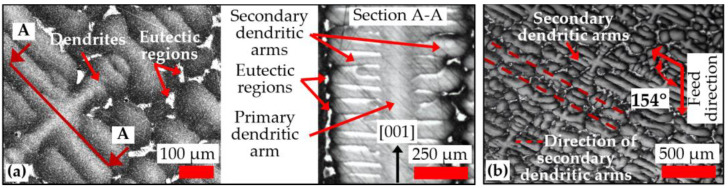
Metallographic microstructure of the CMSX-4 Ni-based superalloy highlighting (**a**) the orientation of dendritic growth and (**b**) the feed direction relative to the secondary dendritic arms.

Slot-milling experiments were undertaken on a Matsuura LX-1, 3-axis linear motor, high-speed machining (HSM) centre with a maximum spindle power of 4.5 kW and rotational speed range of 200–60,000 rpm. The workpiece material was a single-crystal CMSX-4 Ni-based superalloy processed by investment casting with a hardness of ~41 HRC and nominal chemical composition as detailed in Table 1. Rectangular blocks (65 × 50 × 6 mm) were cut out from larger cast test pieces using wire electrical discharge machining (WEDM). The surfaces of the rectangular workpieces were then machined square by subsequent face-milling operations. Figure 1 shows optical micrographs (imaged using a Leica light-polarising microscope) of the representative microstructure of the single-crystal alloy, which was revealed by the mounting, grinding and polishing of a cross-sectioned sample followed by etching in Kalling’s No. 2 reagent for approximately 13 s. The workpiece microstructure comprised a network of primary and secondary dendrite structures and eutectic regions, as shown in Figure 1. During the material solidification process, the direction of primary dendrite growth was consistent with the [001] orientation of the seed (grain selector); see Figure 1a. Elements such as Co, Re, W, Cr and Mo tend to concentrate in the dendritic core, whilst others such as Ti, Al and Ta segregate towards the interdendritic regions [35]. As the microstructure of single-crystal alloys are known to demonstrate heterogeneous/directional characteristics [36], the tool feed direction in all tests was aligned at an angle of ~154˚ relative to the orientation of the secondary dendritic arms, as illustrated in Figure 1b.

**Figure 2:**
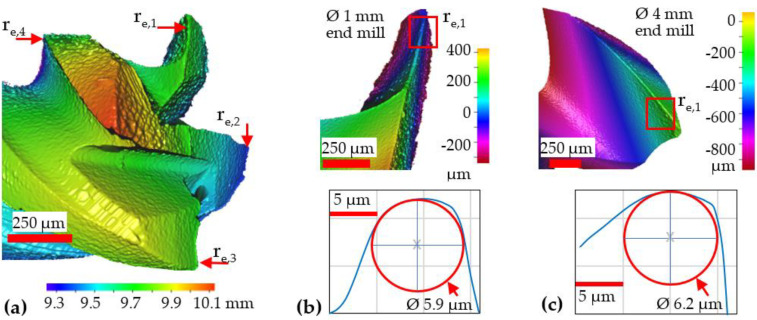
(**a**) Sample 3D scan of tool surface highlighting the measured cutting-edge radii, with corresponding measured sample edge profiles for (**b**) Ø 1 mm and (**c**) Ø 4 mm end-mills.

A full-factorial experimental design was carried out, incorporating two variable factors encompassing 2 different tool diameters (1 and 4 mm) and 4 feed rate levels (25, 100, 175 and 250 mm/min). All of the cutting tools evaluated were supplied by Seco Tools (JS514010F2C.0Z4-NXT and JS514040F2C.0Z4-NXT), which were 4-fluted, TiAlN-coated tungsten carbide (WC) end-mills with equivalent geometries including helix and rake angles of 46˚ and 8˚, respectively. Due to limitations in workpiece material availability, each test was performed once without any replication. Prior to test commencement, the cutting-edge radii (r_e_) of each flute on all tools were scanned and measured using an Alicona InfiniteFocus 3D microscope as demonstrated in Figure 2. Results indicated that the average r_e_ was largely similar for all the end-mills regardless of diameter and was in the region of 6.0 ± 0.3 μm. Cutting speed (v_c_) and depth of cut (a_p_) were fixed at 126 m/min and 100 μm, respectively, as detailed in the experimental array shown in Table 2 (experimental runs were performed in a randomised order). The feed rate (v_f_) levels were selected to ensure that the corresponding range of uncut chip thicknesses (equivalent to the feed per tooth) largely encompassed the anticipated minimum chip thickness (h_min_) spectrum of 0.2–0.4 r_e_ [9]. Preliminary trials involving the Ø 1 mm end-mills revealed that operating at feed rates higher than 250 mm/min resulted in premature tool failure, which therefore limited the f_z_ to 1.563 µm/tooth (0.26 r_e_)_._ A relatively high cutting speed (126 m/min) was utilised as Gao et al. [34] concluded that the shortened chip deformation time when machining DD98 single-crystal nickel-based superalloy at cutting speeds up to 105 m/min reduced frictional forces at the tool–chip interface and improved workpiece surface roughness. The end of test/tool life criteria were a maximum flank/rake face wear of 100 and 300 µm for the Ø 1 mm and Ø 4 mm end-mills, respectively, or a maximum cutting length (Lc) of 650 mm (10 full immersion slots, each over a length of 65 mm and depth of 100 µm).

**Figure 3:**
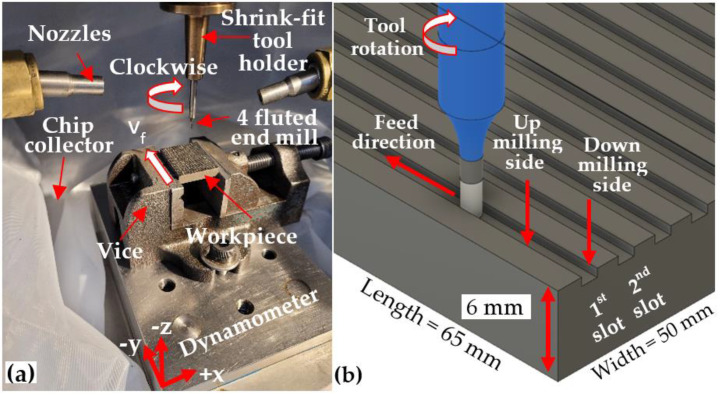
(**a**) Experimental setup and (**b**) schematic of the slot-milling operation.

The experimental setup and a schematic of the slot-milling operation are shown in Figure 3a,b, respectively. The workpiece blocks were held in a vice mounted on a piezoelectric three-component platform dynamometer (Kistler 9257A), which was clamped onto the machine worktable. The cutting fluid employed was a Hocut 3380 water-based emulsion with 7% concentration mineral oil supplied externally via two opposing nozzles at low pressure (< 2 bar) and flow rate of ~21 L/min. Cutting tools were held in MST Mizoguchi HSK shrink fit toolholders. A polypropylene filter bag with a 10 µm mesh was placed between the dynamometer base and spindle, which was used as a sieve to trap and retain chips generated during the tests.

**Figure 4:**
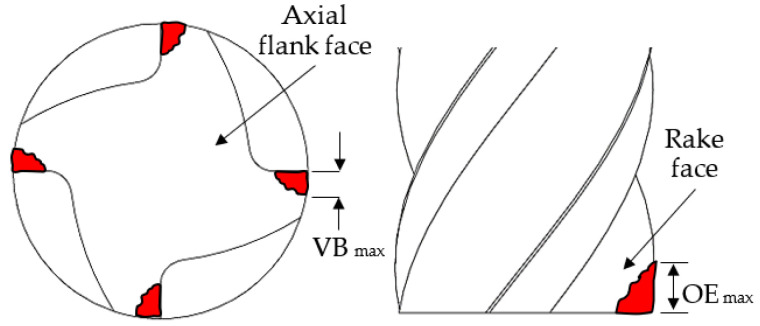
Schematic representation of measured types of tool wear (adapted from [22,37]).

Chip morphology was assessed based on 20 swarf samples collected following each test using the Alicona InfiniteFocus microscope. In order to prevent the cross-contamination of chips between different tests, the machine tool work area was thoroughly cleaned before the start of each trial. Tool wear at the axial flank and rake faces was measured at regular cutting length intervals (at least five readings were recorded with the specific intervals dependant on wear rate of the respective tools) using an optical toolmaker microscope equipped with an X–Y linear micrometre stage and attached Nikon EOS digital camera for capturing micrographs of wear scars. Figure 4 illustrates the types of tool wear measured according to ISO8688 [37] and Alhadeff et al. [22], with the maximum wear length at each cutting edge recorded and then averaged. For the Ø 4 mm tools, both the maximum outside edge (OE_max_) and flank (VB_max_) wear levels were typically discernible. However, due to focal limitations of the toolmaker microscope, there were difficulties in obtaining accurate wear measurements on the rake face for the Ø 1 mm end-mills, as the wear scars generally extended into the curved profile/section of the flutes. Therefore, only flank wear was evaluated for the Ø 1 mm tools.

Force signals from the dynamometer were post-processed using Kistler Dynoware software to determine the root mean square (RMS) values of the resultant cutting force measured over the total length of each machined slot. A low pass filter with a cut-off frequency of 3.5 kHz was applied in order to attenuate force signals approaching and above the resonance frequency of the dynamometer (>4.0 kHz). Specific cutting forces (k_c_) were calculated using Equations (1) and (2) as proposed by Tschätsch [38]:

(1) $$k_{c}=\frac{F_{R}\cos\lambda }{h_{m}\cdot a_{p}}$$

(2) $$h_{m}=\frac{360}{\pi \varphi }\cdot \frac{a_{e}}{\emptyset }\cdot f_{z}\sin\left(90-\lambda \right)$$

where F_R_ is the resultant cutting force, h_m_ is the average chip thickness, φ is the swept angle (180), a_e_ is the radial depth of cut (1 or 4 mm for the Ø 1 mm and Ø 4 mm end-mills, respectively) and λ is the tool helix angle (46).

The areal arithmetic mean roughness (Sa) of the machined surfaces was evaluated according to the ASME B46.1-2019 standard using the Alicona InfiniteFocus microscope. The samples were assessed by scanning an area of 4 × 0.8 mm^2^ at four different locations along the slot length, using an optical magnification factor of 20 with lateral and vertical resolutions of 90 nm and 2.94 μm, respectively, and a cut-off length of 0.8 mm. Optical micrographs of burrs formed on the edges of the machined slots were obtained using the toolmaker microscope to determine the average burr widths by taking the maximum values of 4 measurements at the entrance (Lc = 0–30 mm) and exit (Lc = 30–65 mm) regions of the first slots, as well as at the exit section of the last slots (Lc = 585–665 mm).

## 3. Results and Discussion

### 3.1. Chip Formation

**Figure 5:**
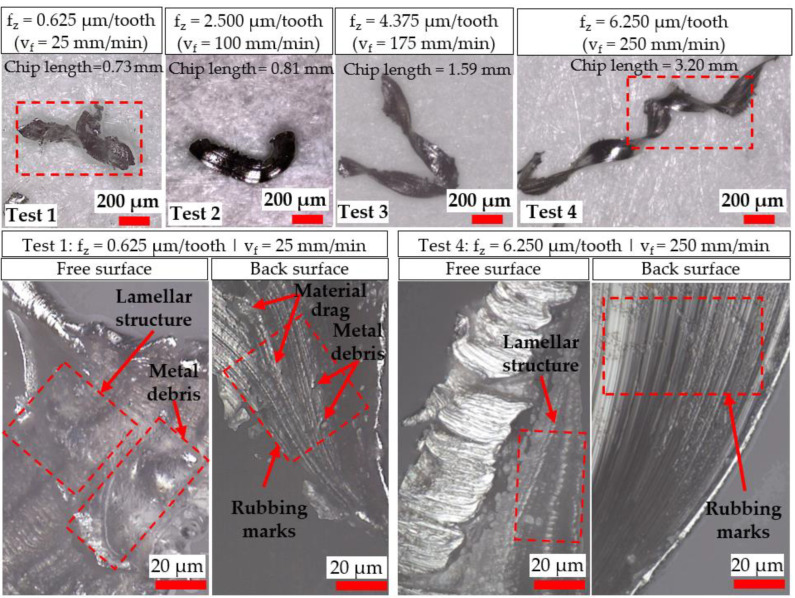
Evaluation of chip morphology from tests performed using Ø 4 mm end-mills.

**Figure 6:**
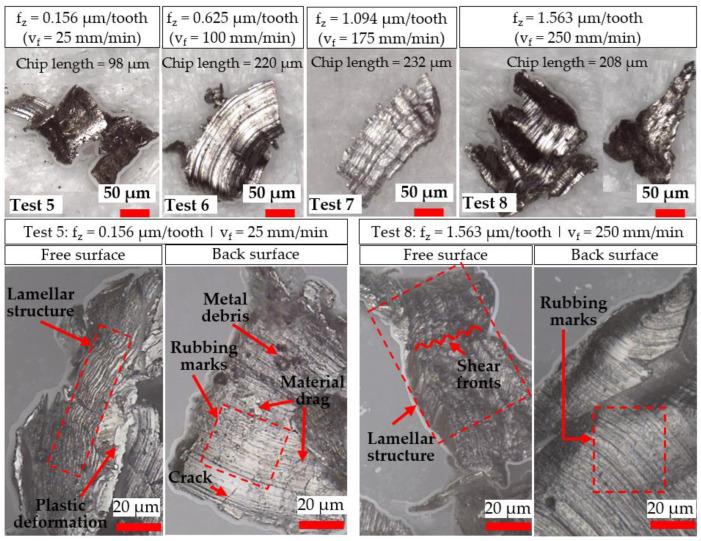
Evaluation of chip morphology from tests performed using Ø 1 mm end-mills.

Images of representative chips from tests using the Ø 4 mm and Ø 1 mm end-mills are presented in Figure 5 and Figure 6, respectively. When milling with the Ø 4 mm end-mills, increasingly longer (0.73–3.20 mm) and curled chips were typically produced with higher f_z_ (0.625–6.250 µm/tooth). When machining at the lowest feed rate level (25 mm/min) with the Ø 4 mm end-mill in Test 1 (f_z_/r_e_ ratio of 0.104), a distinct variation in chip morphology was observed when compared against those from Tests 2–4, with clear signs of material drag, build-up and deformation on the chip surfaces in the former. This, together with the lack of curling or formation of longer helical chips due to premature breakage/separation from the workpiece, suggests the incidence of heavy material ploughing during cutting [24] resulting from the size effect when operating below the minimum chip thickness threshold. In contrast, differences in chip length were marginal (208–230 µm) for tests involving the Ø 1 mm end-mills when f_z_ was varied from 0.625 to 1.563 µm/tooth (Tests 6–8). However, considerably shorter chips (~98 µm) were obtained in Test 5 when cutting with the lowest feed rate of 25 mm/min (0.156 µm/tooth), which corresponded to an f_z_/r_e_ ratio of < 0.03. Furthermore, fragmented and irregular-shaped swarf were typically generated when milling with the 1 mm-diameter tools, indicating that the chips were subjected to relatively high mechanical stresses, most likely the result of ploughing caused by the feed per tooth being considerably lower than the tool cutting edge-radius (f_z_/r_e_ < 0.26).

In general, the free surfaces of the chips were characterised by lamellar-like structures similar to a wave-front texture, generated during shearing of the material. The back surface of the chips typically showed a reduction in material drag marks and metal debris as the feed rate increased. In particular, increased plastic deformation was evident on the chips from Test 5, where a higher density of ‘shear fronts’ on the lamellar structure of the free surface was observed, suggesting material ploughing within this region was prevalent. Additionally, the back surface revealed the presence of cracks, which was attributed to the formation of more fragile and thinner chips, coupled with the dominance of the size effect on the material-removal mechanism (considerable workpiece ploughing likely leading to higher mechanical stresses on the chips).

### 3.2. Tool Wear

**Figure 7:**
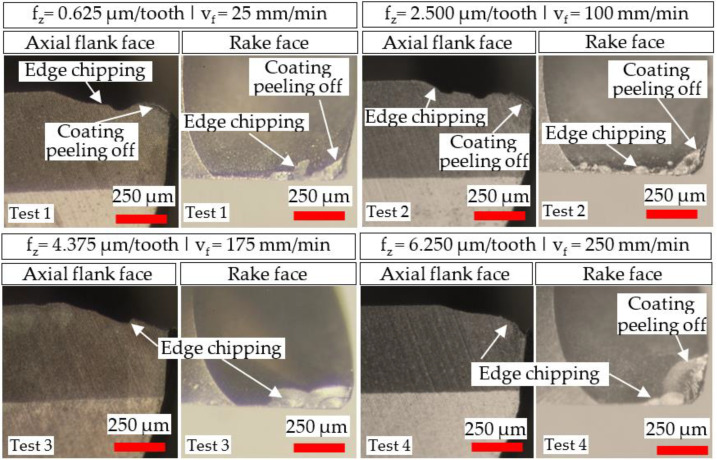
Tool wear micrographs for the Ø 4 mm end-mills at test cessation.

**Figure 8:**
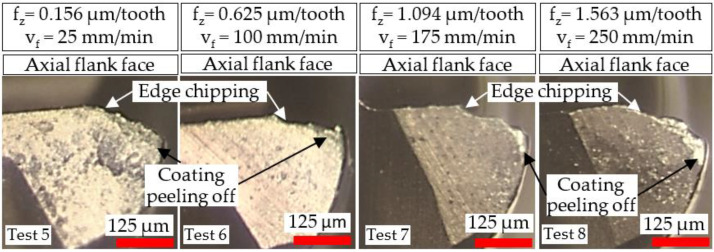
Tool wear micrographs for the Ø 1 mm end-mills at test cessation.

Micrographs of tool wear at the cessation of each test involving the Ø 4 and Ø 1 mm end-mills are presented in Figure 7 and Figure 8, respectively. All of the tools sustained edge chipping together with abrasive wear, which also caused peeling/delamination of the TiAlN coating layer. Similar tool wear modes/mechanisms were reported in previous studies on milling of Ni-based superalloys [39,40]. A noticeably larger wear region was apparent on the rake face of the Ø 4 mm end-mill used in Test 4 (v_f_ = 250 mm/min), which was attributed to a combination of the higher chip load (6.250 µm/tooth) and longer chips produced, as shown in Section 3.1.

Compared to the Ø 4 mm end-mills, a considerably greater loss of the corner edge regions was evident in the Ø 1 mm tools. This was probably triggered by the lower stiffness and smaller corner radius (0.01 mm) of the micro-end-mills, making them more prone to deflection and edge fracture coupled with the increased loading frequency stemming from the higher rotational speed (40,000 rpm).

**Figure 9:**
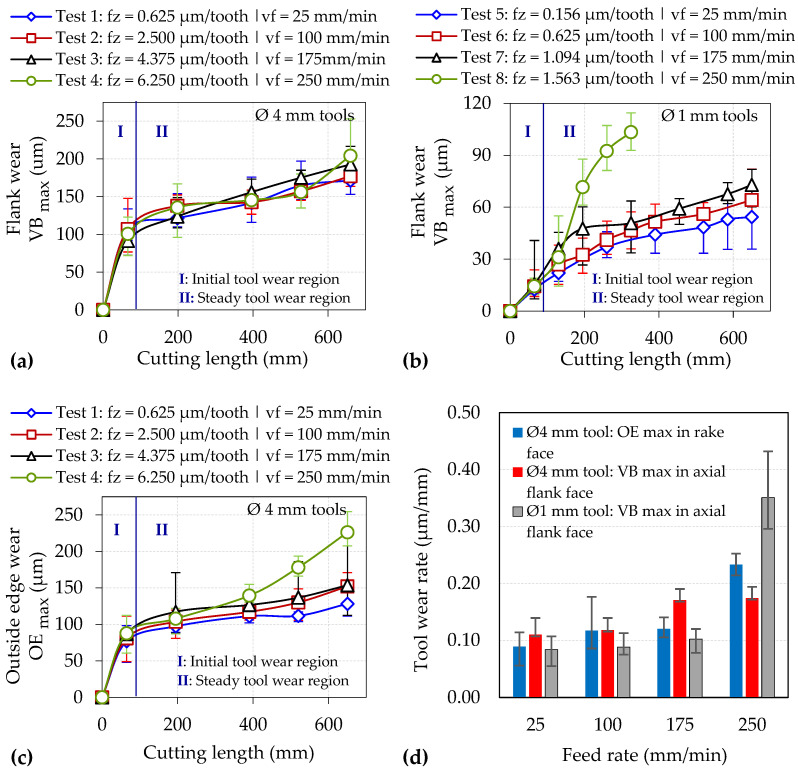
Flank wear curves for the (**a**) Ø 4 mm and (**b**) Ø 1 mm end-mills; (**c**) outside edge wear curves for Ø 4 mm end-mills; (**d**) tool wear rates obtained in the steady wear region (Lc > 90 mm).

The wear progression for both the Ø 4 mm (flank and outside edge) and Ø 1 mm (flank) tools together with the corresponding wear rates are outlined in Figure 9. All of the tools achieved a cutting length of 650 mm without exceeding the respective wear length criterion except for Test 8, which recorded an average maximum flank wear of ~100 µm after machining a distance of ~300 mm. In general, the flank and outside edge wear plots for the Ø 1 mm and Ø 4 mm end-mills shown in Figure 9b,c, respectively, revealed higher tool wear with increasing v_f_, whilst this trend was less apparent in the flank wear results of the Ø 4 mm tools; see Figure 9a. The latter was possibly due to the incidence of abrupt wear/fracture of the cutting edges at various stages over the test duration. Furthermore, a steeper rise in wear levels was apparent following the initial stages of tests involving the highest feed rate of 250 mm/min (Ø 4 mm: 6.250 µm/tooth vs. Ø 1 mm: 1.563 µm/tooth), as exemplified by the significantly accelerated wear rates of up to 0.23 µm/mm (rake face) and 0.35 µm/mm (axial flank face) for the Ø 4 mm and Ø 1 mm tools, respectively; see Figure 9d.

### 3.3. Cutting Forces

**Figure 10:**
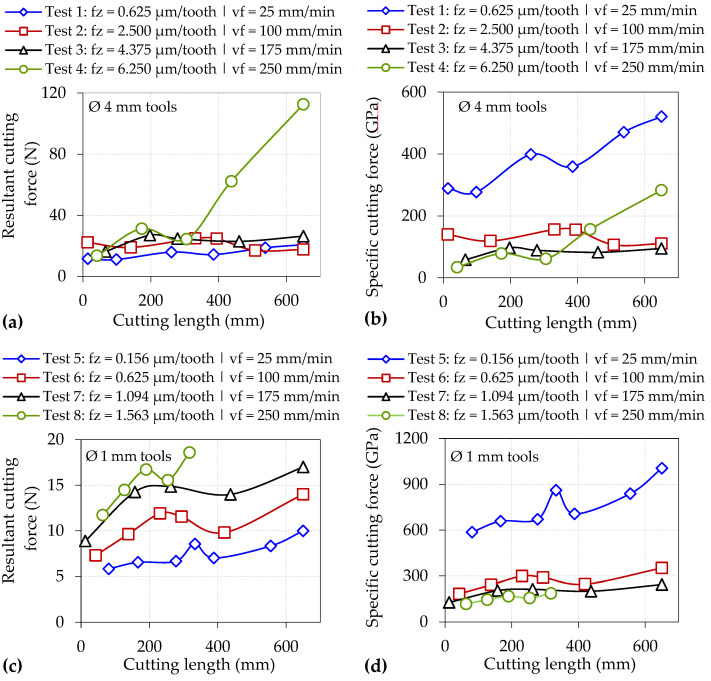
Resultant and specific cutting forces for tests using: (**a**,**b**) Ø 4 mm and (**c**,**d**) Ø 1 mm end-mills.

The root mean square (RMS) values of the resultant and specific cutting forces recorded with respect to cutting length for each test are presented in Figure 10. All of the trials involving the Ø 4 mm tools showed comparable resultant force levels ranging between ~15–30 N that remained relatively stable throughout the test duration except for Test 4, which revealed a more than four-fold increase from ~25 N after a cutting length of 300 mm to nearly 120 N at test cessation. This was attributed to the rapid rise in tool wear on the rake face measured over the corresponding period from ~125 µm to ~230 µm, as outlined previously in Section 3.2; see Figure 9c. Here, the severely worn rake face likely hindered the flow of the chip along the secondary shear zone, thereby causing an increase in frictional forces. In contrast, forces generated by the Ø 1 mm cutters generally registered an upward trend with increasing cutting length, especially when machining at higher feed rates. However, none of the cutting forces exceeded 20 N even at test cessation (see Figure 10c) despite substantial flank wear on the end-mill used in Test 8, as shown in Figure 9b. In this case, a sharp increase in forces was not observed as occurred with Test 4, suggesting that the significantly shorter chip associated with the Ø 1 mm end-mills led to similar tool wear on the rake face for all tests, regardless of the feed rate.

A characteristic indicator signalling the onset of the size effect is a large increase in specific cutting forces despite a decrease in uncut chip thickness [9]. For the Ø 4 mm end-mills, specific cutting forces were largely under 200 GPa when operating at feeds ≥ 2.50 µm/tooth (except towards the end of Test 4), whereas considerably higher levels ranging from ~300 to over 500 GPa were observed when machining at the lowest feed of 0.625 µm/tooth in Test 1; see Figure 10b. Similarly, Figure 10d shows the specific cutting forces surpassing 600 GPa when machining at 0.156 µm/tooth using the Ø 1 mm cutters (Test 5), in contrast to values below 400 GPa for tests at higher feed rates (Tests 6–8). The significantly higher specific cutting forces recorded in Test 1 and Test 5 were consistent with the different chip morphology previously shown in Figure 5 and Figure 6, respectively, indicating that material ploughing was predominant during chip formation in these trials. Furthermore, specific cutting forces were generally up to two times higher when milling with the Ø 1 mm end-mills as opposed to the Ø 4 mm cutters at equivalent feed rates due to the lower f_z_/r_e_ ratio of ≤ 0.26 in the former, highlighting that the influence of the size effect was more prevalent in the trials using the smaller tools. However, when comparing the results from Test 1 against Test 6 (both undertaken at the same f_z_ of 0.625 µm/tooth and therefore have comparable uncut chip thicknesses), the specific cutting forces were relatively higher (521 vs. 352 GPa) when using the larger end-mill. This was attributed to the extended arc length traversed by the cutting edges, leading to higher forces (26 vs. 14 N) as a result of the greater volume of material removed and longer chips, formed predominantly through ploughing rather than shearing as discussed previously in Section 3.1.

### 3.4. Machined Surface Quality

**Figure 11:**
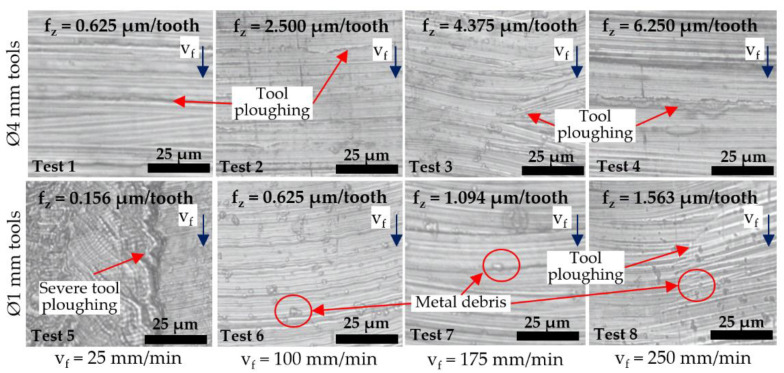
Optical micrographs of machined surfaces at the end of each test.

**Figure 12:**
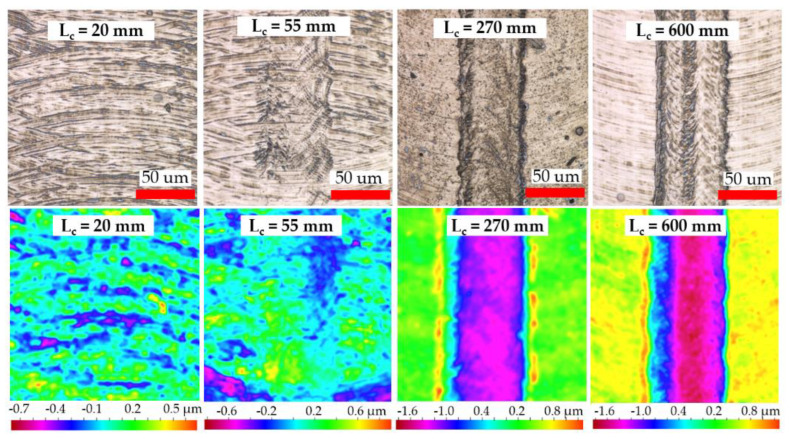
Micrographs and contour plots showing progression of material ploughing and depth of grooving for slots machined in Test 5. v_f_ = 25 mm/min, f_z_ = 0.156 µm/tooth.

**Figure 13:**
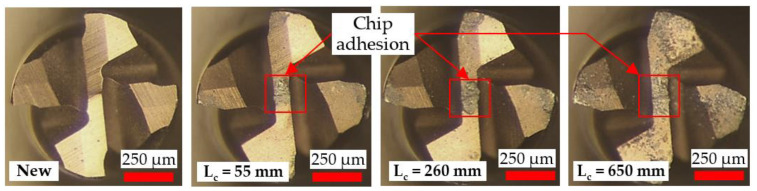
Micrographs of tool wear progression in Test 5. v_f_ = 25 mm/min, f_z_ = 0.156 µm/tooth.

Figure 11 shows optical micrographs of representative slot surfaces machined with worn tools at the end of each test. Minor tool ploughing marks and adhered material/chips were evident on the majority of the machined surfaces, which likely resulted from a combination of the relatively small f_z_ employed and the progressive loss of the tool cutting edge. The only exception was in Test 5 (Ø 1 mm tool at 0.156 µm/tooth), where the rapid progression of material ploughing and groove formation in the middle of slots extending to a depth of up to ~3 µm was observed after a cutting length of 55 mm; see Figure 12. This was the result of significantly higher specific cutting forces and adverse chip morphology contributing to material adhesion/build-up on the centre region of the end-mill (see Figure 13), which caused an undesired interaction with the workpiece.

**Figure 14:**
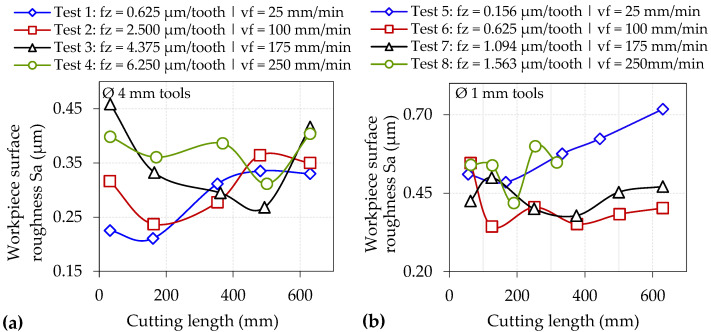
Workpiece surface roughness of slots machined using (**a**) Ø 4 mm and (**b**) Ø 1 mm end-mills.

The results of slot surface roughness measurements against cutting length for the Ø 4 mm and Ø 1 mm tools are displayed in Figure 14a,b, respectively. In general, lower surface roughness levels were recorded when utilising the larger end-mills (0.21–0.46 µm Sa) as opposed to the smaller diameter tools (0.35–0.72 µm Sa), despite the former employing higher values of feed per tooth. This can be attributed to the larger contact region of the Ø 4 mm end-mills with the machined surface coupled with the greater incidence of the size effect in the tests using the Ø 1 mm tools, resulting in smaller, fragmented chips, which were more prone to adhere onto the workpiece surface.

The surface roughness curves for all tests utilising the Ø 4 mm end-mills exhibited no obvious trend with increasing cutting length/tool wear progression. Instead, the Sa values were seen to fluctuate, most likely due to edge fracture/wear of the end-mills. Likewise, there was no discernible correlation between the surface roughness results and cutting length in tests involving the Ø 1 mm end-mills operating at feed rates of 100 to 250 mm/min (Tests 6–8). However, a clear upward trend in surface roughness was observed with increasing cutting length/tool wear in Test 5 when machining at the lowest feed rate of 25 mm/min, which corresponds to the increasing level of ploughing and groove formation in the slots shown previously in Figure 12.

### 3.5. Burr Formation

**Figure 15:**
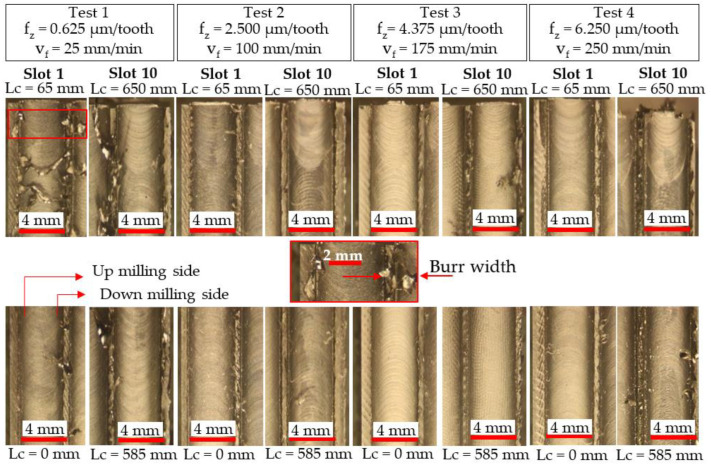
Optical micrographs of burr formation on the first and last slots machined using Ø 4 mm end-mills.

**Figure 16:**
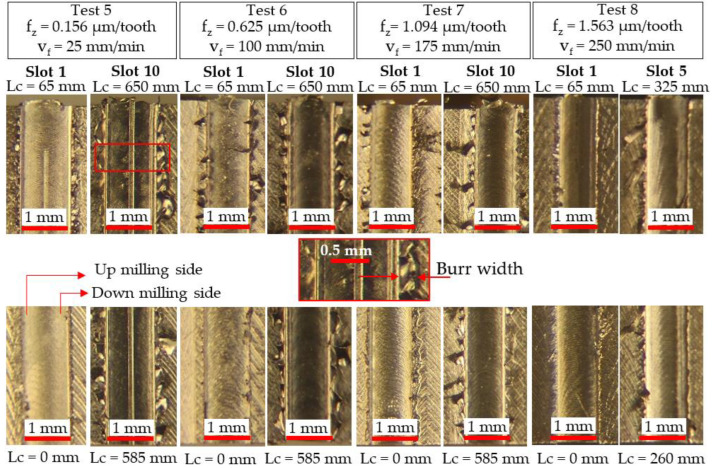
Optical micrographs of burr formation on the first and last slots machined using Ø 1 mm end-mills.

**Figure 17:**
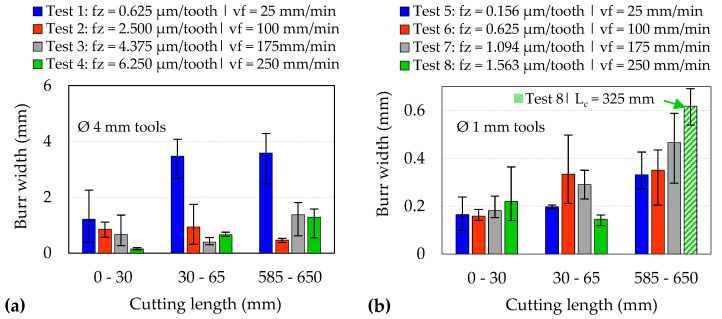
Average burr width for slots machined using (**a**) Ø 4 mm and (**b**) Ø 1 mm end-mills.

Figure 15 and Figure 16 detail optical micrographs of burr formation around the entrance and exit location of the first and last slots machined using the Ø 4 mm and Ø 1 mm end-mills, respectively, while corresponding measurements of average burr width are shown in Figure 17. When machining with new Ø 4 mm end-mills, shorter burrs were apparent at test commencement (Lc < 30 mm) on the sides cut under up-milling mode. As the cutting-edge transitioned to down-milling commencing at the centre of the slot, the chip thickness reduced from a maximum value to zero at the end of the tooth–workpiece engagement, leading to diminished shearing but greater material side flow. Hence, larger burrs were observed on the down-milling side of the slot [41]. However, the discrepancy between the up- and down-milled sections became less apparent as the level of burring increased with the progression of tool wear, which led to higher chip extrusion and ploughing at both edges of the slot walls. In Tests 2–4 (Ø 4 mm tools), the average maximum burr width did not exceed 1.5 mm, even for slots machined with tools in the worn condition. In contrast, considerably larger burrs (~3.6 mm) were generated even on the first slot in Test 1, which further demonstrates the likely prevalence of material ploughing during chip formation when cutting with a low feed rate (25 mm/min), as the f_z_/r_e_ ratio was ~0.1.

For slots machined with the Ø 1 mm end-mills, the burr size was generally found to increase with tool wear/cutting length. The level of burr formation, however, was generally comparable on both sides of the slots, possibly as a result of the low uncut chip thickness values employed (f_z_: 0.156–1.560 µm/tooth). In terms of burr width, the largest values were observed on the last slot machined in Test 8 (~0.62 mm), which corresponded to the highest tool wear level as shown previously in Figure 9b. However, burrs were seen to continuously form on both edges along the entire length of slots machined in Test 5 (v_f_ = 25 mm/min l f_z_ = 0.156 µm/tooth), resulting from the influence of the size effect.

## 4. Conclusions

Based on the analysis of chip formation and specific cutting force data, the minimum uncut chip thickness when micromilling CMSX-4 single-crystal Ni-based superalloy using Ø 1 mm tools was estimated to lie within 0.03 r_e_ < h_min_ < 0.10 r_e_. This is considerably lower than the h_min_ values for polycrystalline alloys, which are commonly reported in the literature as ranging from 20% to 40% of r_e_. Elevated specific cutting forces of up to ~ 1000 GPa together with uncharacteristically short (~98 µm) and erratically formed swarf were observed when machining at the lowest feed rate of 25 mm/min (f_z_ = 0.156 µm/tooth) using the micro-end-mills. This was likely caused by heavy ploughing that promoted chip adhesion on both the cutting tools and workpiece, which induced deterioration of the slot surface roughness by up to 35% (from ~0.4 to 0.7 µm Sa). Additionally, burrs were continuously generated along the entire length of the slot walls, although the variation in burr widths as the feed rate increased was reasonably moderate. Despite having similar cutting-edge radii of ~6 µm, the results from the experiments involving the larger Ø 4 mm end-mills appeared to suggest a change in the onset of the size effect, with a larger corresponding minimum chip thickness approximated at 0.10 r_e_ < h_min_ < 0.42 r_e._ This was due in part to the longer tool–workpiece contact arc, which resulted in irregularly shaped chips with average lengths of ~730 µm, together with a noticeable increase in specific cutting forces above ~500 GPa and considerably larger burrs (~3.6 mm), when operating at 25 mm/min (f_z_ = 0.625 µm/tooth). However, varying the feed rate from 25 to 250 mm/min had a negligible effect on the workpiece surface roughness. The incidence of the size effect, however, did not have a significant detrimental influence on tool wear. Indeed, higher wear rates were obtained with increasing feed rate/uncut chip thickness, in particular the Ø 1 mm tool, when machining at 250 mm/min (f_z_ = 1.563 µm/tooth), resulted in the premature failure of the end-mill.

## Figures and Tables

**Table 1:** Nominal composition (%weight) of single-crystal CMSX-4 Ni-based superalloy.

Cr	Co	Mo	W	Al	Ti	Ta	Re	Hf	Ni
6.5	9.0	0.6	6.0	5.6	1.0	6.5	3.0	0.1	Bal

**Table 2:** Test parameters and levels.

Test No.	Tool Diameter, Ø (mm)	Cutting Speed, v_c_ (m/min)	Feed Rate, v_f_ (mm/min)	Feed per Tooth, f_z_ (μm/Tooth)	f_z_/r_e_
1	4	126 (10,000 rpm)	25	0.625	0.104
2			100	2.500	0.417
3			175	4.375	0.729
4			250	6.250	1.042
5	1	126 (40,000 rpm)	25	0.156	0.026
6			100	0.625	0.104
7			175	1.094	0.182
8			250	1.563	0.261

## Data Availability

Not applicable.
